# Medical Items and News of Interest to the Physician

**Published:** 1884-01

**Authors:** 


### For the Use of Physicians.

CULLED FROM THE STANDARD MEDICAL JOURNALS OF THE WORLD.

NOTE.—These formulæ are intended only for the regular practitioner of medicine. and should be employed only by him, or under his immediate supervision.

**Typhoid and Intoxication.**

Murchinson says that when alcohol intoxicates a typhoid fever patient the fact indicates his convalescence.—*Med. Age.*

**Gastric Ulcer.**

℞. Bismuth sub. nit., magnesiæ carb., āā gr. xv.; liq. morph. hydrate, ♏ xv.; aquæ ad., ʒ j. M.—*Dr. Andrew Clark.—New Eng. Med. Mo.*

**Gastralgia.**

In gastralgia, Professor Bartholow recommends atropine in the dose of $\frac{1}{200}$ grain hypodermically, by enema or in the form of suppositories.—*Med. Herald.*

**Tonsilitis.**

Dr. Isaac Barton, in a very able paper before the Philadelphia Laryngological Society, called attention to the very good results achieved in tonsilitis by an injection into the tonsil of a glycerole of ergot.—*Med. Bulletin.*

**Malarial Hematuria.**

In a well marked case of this disease, complicating Bright's disease, the hemorrhage was promptly arrested by teaspoonful doses of quina phenate, hourly, in a half teacup of warm tea.

**Syphilis and Vaccinia, Antagonism Between.**

In the *Gazette Hebdomadaire,* M. Polin, a public vaccinator in one of the military districts, states that in nearly every instance in which a child suffers from hereditary syphilis, vaccination will fail.—*Med. News.*

**Cancer.**

Dr. Brandini, of Florence, has recently discovered that citric acid will assuage the violent pain which is the usual concomitant of cancer. He applies to the part pledgets of lint soaked in a solution of four grains of the acid in 350 grains of common water, with the result of affording instantaneous relief in the most aggravated cases.—*Galignani's Mes.—N. Y. Med. Times.*

**Erysipelas.**

Dr. Flaminio Tassi, of Siena, has published a report of four cases of erysipelas, which rapidly yielded to a saturated aqueous solution of picric acid, applied morning and evening with a camel hair brush.—*Med. and Surg. Rep.*

**Spasmodic Cough.**

Dr. William Squire recommends a solution of bromic ether in water (1 to 200) for administration in whooping cough, as well as for angina and spasmodic pain. It may be given in the same manner as the aqua chloroformi of the British Pharmacopœia.—*Med. Times.*

**For Imperfection in Speech.**

Dr. Post, New York, records the case of a girl aged six, who had difficulty in uttering the labial sounds. The mother directed attention to unusual shortness of the frænum of the upper lip, which was divided, with marked improvement.—*N. Y. Med. Jour.*

**Winter Eczema.**

For the relief of winter eczema, a troublesome itching affection, Dr. Squibb's Ephemeris recommends the following: Take of tannic acid, forty grains; of glycerine and alcohol each half a fluid ounce, water to make four ounces. This solution is applied to the itching surfaces by means of a small sponge or rag, morning and evening.—*Detroit Lancet.*

**Zoster, Oil of Peppermint in.**

Dr. Meredith says that he has found the oleum menthæ pip. more effective than any other form of anodyne application he has tried in allaying the neuralgic pains so often piteously complained of in cases of herpes zoster. He has painted the oil over the eruption when it was out in a fresh florid condition, and with great relief to the patient.—*Med. Record.*

**Tin Poisoning.**

An English chemist who had been called upon to analyze several socks and stockings of a red color, which had been found to cause great irritation to the skin of the wearer, discovered the cause of the trouble in the tin salt used as a mordant in fixing the dye. He succeeded in obtaining over twenty-two grains of tin in the form of the dioxide. When acted upon by acid prespiration the tin oxide forms an exceedingly irritating compound.—*Amer. Med. Weekly.*

**Condylomata.**

The following powder is recommended as a specific for the removal of condylomata:

℞.

Hydrarg. chlorid. mit., ʒ i.

Acid. borac., gr. x.

Ft. pulv.—*Med. Times.*

**Infectious Diseases.**

Dr. Walfroid (*Lancet*) draws attention to the great value he attaches to the use of arsenic as a preventative agent in exposure to infectious diseases. Dr. Walfroid believes that by the administration of this drug in any of the diseases of this class during the incubation period, an attack may be prevented or greatly modified.—*New Eng. Med. Mo.*

**Kissing, Danger of.**

Dr. Payne, in the *North Carolina Medical Journal*, strongly condemns kissing, from its liability to propagate disease, and cites the case of a young man in the secondary stage of syphilis conveying the disease, by kissing, to a child sixteen months old, the child in turn giving it to the mother.—*Obst. Gaz.*

**Chalky Deposits.**

Dr. Theo. M. Kendall writes to the *Lancet* that he derived most gratifying results in a case of severe chalk gout, from the use of a lotion of ten grains of salicylate of soda to the ounce. By its use, chalky deposits in the ear were softened, and in four days disappeared, leaving only a small scar.—*Med. and Surg. Rep.*

**Syphilis.**

A solution of iodoform (6 parts) in glycerin (20 parts), of which from 30 to 75 centigrammes are used (gradually increasing) each time, is recommended by E. Thoman for hypodermic injection in intense syphilis. After six to twelve injections he always noticed a great amelioration in the symptoms.—*Bull. de Ther. Med. Times.*

**Weak Heart—Electricity vs. Chloroform.**

A fact of the greatest importance, practically—especially with reference to the treatment of threatening death by chloroform—has just been determined by Professor von Ziemssen. In investigating the effect of electricity upon the heart, he has discovered that the induced current has no influence whatever upon the frequency or force of the cardiac contractions, whilst the continuous or battery current most distinctly affects them.—*Med. Record.*

**Albuminuria.**

In a letter to the *Lancet*, Mr. V. G. Webb states that some five years ago, having ordered full doses of hydrocyanic acid to allay vomiting for a patient with diphtheria, he found the next day that the percentage of albumin in the urine was reduced one-half ; also that he has lately found the drug equally beneficial in scarlatinal nephritis.—*N. Y. Med. Jour.*

**Trichinosis Nodules.**

M. Rathery, in *Le Journal de Med.*, describes the case of a man who had numerous subcutaneous nodules of the size of peas, situated exclusively on the supradiaphragmatic parts of the body. On excising one of these tumors it turned out to be a trichinosis cyst. The patient had never suffered from any general or local symptoms of trichinosis.—*Med. Record.*

**Scars.**

The Boston *Journal of Chemistry* claims that the following mixture placed upon a granulation surface will prevent the scars from appearing at all unsightly, and in fact at times preventing them from being noticeable: Take of borax an ounce and a half, of salicylic acid twelve grains, glycerine three drachms, rose water six ounces; make a solution.—*Chicago Med. Rev.*

**Spasms of the Œsophagus.**

In the *Union Medicale* for October 12, 1882, M. Courtade reports a case which seems to show that a blow on the head or thorax in an individual showing before then no disturbance of the nervous system may be followed by persistent spasm of the œsophagus. In the case reported, bromide of potassium in large doses and repeated catheterization were sufficient to produce a cure.—*Med. News.*

**Whooping Cough.**

M. Dujardin Beaumetz recommends the combination of the bromides and chloral as being very useful in whooping cough. He gives one dessert spoonful of the mixture in a glass of milk, to which the yolk of an egg has been added, evening and morning.

℞

Potassii bromidi, ʒ ss;

Sodii bromidi, ʒ j;

Ammonii bromidi, ʒ ss.

Syr. chloral, ℥ iss.

Aquæ, ℥ ij.

—*Can. Lancet.*

**Secondary Syphilis.**

The following is useful in secondary syphilis, psoriasis, etc.:

℞.

Liq. hydriodatis arsenici et hydrarg., ♏ xxx.

Tinct. zingiberis, ℨ i.

Aquæ, ℥ i.

M. Make a draught to be taken twice a day directly after meals.—*New Eng. Med. Mo.*

**Hæmoptysis.**

℞

Dextro-quiniæ, ℨ j.

Ergotinæ, ℨ j.

Digitalis pulv.,

Ext. hyoscyam, āā grs. v.

M. Ft. pil no xl.

Sig.—Two pills every two or three hours—in epistaxis, hæmoptysis, etc.—*Mo. Rev. Pharm.*

**Mercurial Poisoning.**

The *Medical Times and Gazette* notes several cases of mercurial poisoning (gums swollen, spongy, and tender, with salivation), occurring among men who were employed in exhausting the little globes used in the incandescent system of electric lighting. The poisoning must have been due to mercurial vapor arising from the exhausting pumps, as no mercury was used, except that contained in these pumps.—*Druggist's Cir.*

**Lumbricoides.**

Dr. Semple cites cases in which the following mixture effected the rapid evacuation of lumbricoides. He regards it as a most certain anthelmintic:

℞.

Chloroform, ℨ j.

Castor oil, ℥ j.

Croton oil, ♏ j.

Mix well. Dose from ℨ ss. to ℥ ss.—*Practitioner.*

**Ear, Abscess of the Lobule of the.**

Dr. Luigi G. Doane says: C. L., aged 23 years, called at my office for advise and treatment during December, 1881. Mr. L. stated that while at work a gas retort burst, and a section of it struck him upon the ear and produced an abscess, which being opened gave vent to pus and blood, and the ear was dressed with iodoform, ℨ ss; unguenti simplicis, ℥ j. Two days after, patient was discharged.—*Druggists' Cir.*

**Dyspnœa.**

Eulenberg in the *Medicinal Kalendar* for 1883, gives the following formula for administering the active principle of quebracho, which, it has been claimed, may be used with benefit in all forms of dyspnœa without regard to cause:

℞

Aspidospermine, 1 grm. (gr. xv);

Aquæ distillatæ, 50 grms. (f ℥ jss);

Acidi sulphurici, q. s. ad solve. M.

Dose—1 gramme (15 minims), containing 2 centigrammes (gr. $\frac{1}{3}$) of the remedy, or more. —*Le Progre's Med.—Med. Times.*

**Angina Faucium.**

In the *Riv. Clin. di Bologna*, Professor Concato recommends ether spray as an inhalation in sore throat. The patient takes the exit tube of a Richardson's spray producer in his mouth, and sulphuric ether is sprayed against the pharynx for three minutes; this is repeated every three hours. Six cases were cured without other remedies. Each case began with a rigor and a sharp attack of fever; temperature 104° Fahrenheit. There was swelling of the submaxillary gland, and pain and difficulty in swallowing. The tonsils were swollen and protruding.—*Med. and Surg. Rep.*

**Primary Cancer of the Lung.**

Dr. G. W. H. Kemper says: Given a case of obscure lung disease with œdema of the corresponding arm, enlarged superficial veins of the neck, chest or abdomen, and accompanied with dyspnea, and we could scarcely hesitate to declare upon these symptoms alone that the lung was cancerous.

The following is Stoke's summary of the diagnostic remarks: "That in simple cancerous degeneration of the lung the principal physical signs are the gradual diminution of the vesicular murmur without râle, its ultimate extinction, and the signs of perfect solidification. That evidences of perfect solidification are better found in this disease than in any other pulmonary affection." "Also pain of a continued kind; a varicose state of the veins in the neck, thorax and abdomen; œdema of one extremity; rapid formation of external tumors of a cancerous character; expectoration similar in appearance to current jelley; resistance of symptoms to ordinary treatment. That, though none of the physical signs of this disease are (separately considered) peculiar to it, yet *that their combination and modes of succession* are not seen in any other affection of the lung."—*Obst. Gaz.*

**Fetid Bronchitis.**

Under the above title Dr. E. Lancereaux, in a recent issue of the *Bulletin de Therapeutic*, treats of fetid bronchitis chiefly from the therapeutical point of view. The real causes of the malady are not fully understood.

The mode of administering the remedy is very simple. It consists in giving four to five gramms, ( about 62 to 80 grains) in an ordinary solution daily for a month or six weeks, after which the amount used will be determined by the conditions present.—*Med. News.*

---

**Gall Bladder—Excision of the.**

Dr. C. Langerbuch (*Berliner Klinische Woch.*) has successfully excised the gall bladder to prevent the formation of calculi. He makes an incision parallel to the lower border of the liver, joined by an incision parallel to the outer border of the rectus abdominis. The abdominal cavity thus opened, the traverse colon and small intestines are pushed down by a large sponge and the liver elevated so as to bring prominently forward the hepatico-duodenal ligament. The gall bladder is then easily excised. The cystic duct is laid free and ligated with silk in two places—catgut should not be used. Care is taken to avoid wounding the liver, the abdominal wound is then closed and the operation duly finished. —*Amer. Med. Weekly.*

---

**Sewer Gas Poisoning.**

The Philadelphia *Medical Times* contains the report of Dr. J. T. Eckridge of an interesting case of the poisoning of a strong healthy man who went into a privy-well, about fifteen feet deep. for the purpose of cleaning it. Before going in a light had been lowered, but it was not extinguished, and it is important that the fact should be known that this is no sufficient test of the presence of deadly gases in such places. The poisonous gas in this case was probably either sulphureted hydrogen or sulphide of ammonia, or a mixture of the two. Insensibility was rapidly produced, and his condition for several hours was such that death seemed almost inevitable. He was saved by twelve successful injections of aqua ammonia into the superficial veins of the fore-arm; a heroic method of treatment, used only as a last resort after all the usual methods had failed.—*Med. Record,*

**Nocturnal Incontinence of Children.**

This trouble is annoying and often intractable. Prof. S. D. Gross (*Mich. Med. News*) advises the use of the following formula:

℞
Strychnine, gr. i.
Pulv. canthar. gr. ii.
Morphiæ sulph., gr. ij.
Ferri pulv., gr. xx.

He directs the pill mass to be divided into forty or fifty pills, according to circumstances. Under the use of this formula, together with careful hygiene, almost all cases of nocturnal incontinence in children rapidly improve.—*Gaillard's Med. Journal.*

---

**Typhus and Typhoid.**

The most frequent of these, according to Lori (*Jahrbucher fur Kinderheilkunde*), is an acute catarrh of the larynx, pharynx, and trachea. Œdema of the vocal cords sometimes occurs, from which result functional vocal troubles of various kinds. Diphtheritis and laryngeal œdema rarely occur, and somewhat less frequently the paralysis of the larynx, pharynx, and trachea therefrom. These are most frequent in the second stage of the fever, as also are aphonia, hoarseness, and difficulty in breathing. The most frequent and serious complication is laryngeal perichondritis, which usually occurs in the sixth to eighth week of the fevers. The cricoid, arytenoid cartilages, and epiglottis are most frequently affected. The treatment is locally astringents, and internally wine and iron, especially with paralysis.—*Gaillard's Med. Jour.*

---

**Mixed Chloroform.**

This method, as practiced by Prof. Thiersch, in Leipsic, succeeds frequently, without producing unconsciousness, in causing a perfect analgesia, during which Thiersch had made grave and prolonged operations.

To be certain of this narcosis, drunkards receive one-half to one grain morphia; men, one-half of a grain; women one-quarter of a grain, and children one-twelfth to one-eighth of a grain. From five to seven minutes after the hypodermic injection the patients are very lightly chloroformed, till near the stage of excitement; the operation is performed; as soon as pain is felt a little chloroform is added. In this manner not the tenth part of chloroform is needed, the operation just as painless as under full narcosis, and there is no risk or danger incurred.—*Med. and Surg. Rep.*

